# Subjektives Befinden und Arbeitsfähigkeit nach SARS-CoV-2-Immunisierung mit dem Vektor-Impfstoff ChAdOx1-S (AstraZeneca COVID-19–Vakzin): Ergebnisse einer anonymen Umfrage

**DOI:** 10.1007/s40664-021-00448-4

**Published:** 2021-11-07

**Authors:** Johannes Kalbhenn, Feline Gabler, Sebastian Heinrich, Daniel Steinmann

**Affiliations:** 1 Klinik für Anästhesiologie und Intensivmedizin, Universitätsklinikum Freiburg, Hugstetter Straße 55, 79106 Freiburg im Breisgau, Deutschland; 2 Betriebsärztlicher Dienst, Universitätsklinikum Freiburg, Freiburg, Deutschland

**Keywords:** COVID-19, Impfung, Nebenwirkung, Arbeitsunfähigkeit, Personalplanung, COVID-19, Vaccination, Side effects, Incapacity for work, Personnel planning

## Abstract

**Hintergrund:**

Seit dem 29.01.2021 wurde der COVID-19-Impfstoff ChAdOx1‑S (Vaxzevria, AstraZeneca) durch das Paul-Ehrlich-Institut in Deutschland zugelassen. In mehreren Kampagnen wurde MitarbeiterInnen des Gesundheitssystems und Medizinstudierenden die Impfung mit diesem Vakzin auf freiwilliger Basis angeboten.

**Ziel der Arbeit:**

Primärer Endpunkt der Arbeit war die Erfassung der Rate und Dauer von Arbeitsunfähigkeits(AU)-Meldungen von Arbeitnehmern in Folge der Erstimmunisierung mit ChAdOx1‑S. Sekundäre Endpunkte waren Art und Ausprägung von Nebenwirkungen sowie die selbstempfundene Verträglichkeit.

**Material und Methoden:**

Anonymisierter Online-Fragebogen, einmalig ausfüllbar durch alle Geimpften nach Erhalt der ersten Dosis von ChAdOx1‑S. Die Ausprägung von Nebenwirkungen wurde über eine ordinale numerische Rating-Skala mit Werten zwischen 0 und 10 abgefragt. Weitere wesentliche Datenpunkte waren Alter, Geschlecht und Berufsgruppe. Die Arbeitsfähigkeit in den Folgetagen der Injektion wurde ebenfalls durch Selbstangabe erhoben.

**Ergebnisse:**

Es wurden Daten von 1988 Befragten ausgewertet. Das mittlere Alter lag bei 37,13 (13,73) Jahren (Standardabweichung). 69,8 % der Befragten waren weiblich, 48,1 % gehörten zu therapeutischen und technischen Berufsgruppen mit Patientenkontakt, 38 % waren Studierende, 10,6 % waren dem pflegerischen und 4 % dem ärztlichen Dienst zuzuordnen. Nur 14,4 % der Befragten gaben an, die Impfung ohne Nebenwirkungen vertragen zu haben. Häufigste Nebenwirkung war Müdigkeit, gefolgt von Schmerzen an der Injektionsstelle. In absteigender Häufigkeit folgten Kopfschmerzen, Gliederschmerzen und Schüttelfrost. Nach der Impfung fühlten sich 18 % der Befragten unmittelbar wieder arbeitsfähig. 51 % aller Befragten mussten sich nach der Impfung für mindestens einen Tag arbeitsunfähig melden. Nebenwirkungen waren bei männlichen und jüngeren Befragten stärker ausgeprägt.

**Schlussfolgerung:**

Die Impfung mit ChAdOx1‑S führte häufig zu Nebenwirkungen. Diese hatten bei 37 % der Befragten eine Krankmeldung zur Folge. Dennoch würden sich 89,6 % aller Befragten wieder für eine Impfung mit ChAdOx1‑S entscheiden.

## Abstract (en)

**Background:**

The SARS coronavirus 19 vaccine ChAdOx1‑S (Vaxzevria, AstraZeneca) has been licensed since January 2021 by the Paul Ehrlich Institute for Germany. In several campaigns, healthcare workers and medical students were offered this vaccine on a voluntary basis.

**Aim:**

The primary endpoint of the study was to assess the rate and duration of the incapacity to work as a result of initial immunization with ChAdOx1‑S. Secondary endpoints were type and severity of adverse events and self-perceived tolerability.

**Material and methods:**

Anonymized online questionnaire to be completed once by all vaccinated individuals after receiving the first dose of ChAdOx1‑S. The severity of side effects was queried using an ordinal numerical rating scale with values ranging from 0 to 10. Other key data points were age, sex, and occupational group. Ability to work in the days following the injection was also assessed by self-reporting.

**Results:**

Data from 1988 respondents were analyzed. The mean age was 37.13 years (standard deviation 13.7 years). Of the respondents 69.8% were female, 48.1% belonged to therapeutic and technical professions with patient contact, 38% were students, 10.6% were nursing personnel and 4% were physicians. Only 14.4% of respondents reported having tolerated the vaccination without side effects. The most common side effect was fatigue, followed by pain at the injection site. This was followed in descending frequency by headache, aching limbs, and chills. After vaccination 18% of respondents felt able to return to work immediately, 51% of all respondents had to report themselves unfit for work for at least 1 day after vaccination. Side effects were more prevalent in male and younger respondents.

**Conclusion:**

Vaccination with ChAdOx1‑S frequently resulted in side effects. These resulted in 37% of respondents reporting sick. Nevertheless, 89.6% of all respondents would choose coronavirus vaccination with ChAdOx1‑S again.

## Hintergrund und Fragestellung

Gegen SARS-CoV‑2 wurden Impfstoffe mit völlig neuer Wirkweise entwickelt. Diese Vakzine basieren auf Sequenzen aus entweder Desoxyribonukleinsäure (DNS) oder Ribonukleinsäure (RNS), die jeweils für ein Oberflächenantigen des Virus codieren. Körpereigene Zellen im Bereich des Injektionsorts synthetisieren dieses Antigen, welches in der Folge die Immunantwort auslöst. ChAdOx1‑S des schwedischen Konzerns AstraZeneca basiert auf DNS, die in die Hülle eines Adenovirus verpackt ist, der als *Vektor* die Internalisierung in die Körperzelle ermöglicht. Dabei wurde ein Schimpansen-Adenovirus gewählt, um die Wirksamkeit des Vakzins nicht durch Wirtsantikörper gegen humanpathogene Adenoviren abzuschwächen. Das Vakzin enthält keine replikationsfähige DNS des Vektorvirus, sondern ausschließlich ein in Zellkulturen repliziertes Fragment des Coronavirus-Genoms. Zwei Dosen des Vakzins sind für die vollständige Immunisierung nötig. Bei praktisch allen Immunisierten kann die schwere Erkrankung, bei bis zu 79 % sogar die Symptome von COVID-19 verhindert werden. In den Zulassungsstudien klagten etwas über 60 % aller Probanden über milde bis moderate Nebenwirkungen. Neben Schmerzen an der Injektionsstelle wurden am häufigsten Allgemeinsymptome wie Kopfschmerzen, Müdigkeit und Unwohlsein, gefolgt von Muskelschmerzen, Schüttelfrost und Fieber berichtet [1–5]. In einer Observationsstudie an mehreren tausend MitarbeiterInnen des Gesundheitssystems in Südkorea zeigten mehr als 90 % Nebenwirkungen nach Immunisierung mit ChAdOx1‑S. In über 80 % waren es lokale Schmerzen an der Einstichstelle, fast 80 % klagten aber auch über systemische Nebenwirkungen wie Muskelschmerzen, gefolgt von Kopfschmerzen und Schüttelfrost [5]. In einer Observationsstudie an medizinischem Personal einer Universitätsklinik aus Deutschland meldeten sich 65,3 % aller ArbeitnehmerInnen nach der ersten Dosis von ChAdOx1‑S für mindestens einen Tag arbeitsunfähig [6]. Seit Juni 2021 partizipieren auch Betriebsärzte an der Impfkampagne in Deutschland. Angesichts der Nebenwirkungen ist davon auszugehen, dass ein Teil der ArbeitnehmerInnen in Folge der Immunisierung eine temporäre Arbeitsunfähigkeit anzeigt. Dieses hat Implikationen für die Planung von betrieblichen Impfkampagnen. Diese Observationsstudie untersucht daher anhand des Beispiels von Beschäftigten des Gesundheitssystems und an Medizinstudierenden die Rate und Dauer von Krankmeldungen nach Erstimmunisierung mit dem COVID-19-Impfstoff ChAdOx1‑S. Primärer Endpunkt ist die Erfassung der Rate und Dauer von Arbeitsunfähigkeits(AU)-Meldungen von ArbeitnehmerInnen in Folge der Erstimmunisierung mit ChAdOx1‑S. Sekundäre Endpunkte sind Art und Ausprägung von Nebenwirkungen sowie die selbstempfundene Verträglichkeit.

## Studiendesign und Untersuchungsmethoden

Als Erfassungsinstrument für diese Observationsstudie wurde von der Ethik-Kommission der Albert-Ludwigs-Universität Freiburg am 26.01.2021 eine anonyme Umfrage vorgeschlagen und als unbedenklich bewertet. Der Personalrat stimmte dieser Erfassung am 28.01.2021 ebenfalls zu. Die offene Fachschaft Medizin der Albert-Ludwigs-Universität Freiburg sowie das Studiendekanat Humanmedizin unterstützten die Rekrutierung und Datenerfassung bei den Studierenden.

Die Erstellung des Fragebogens erfolgte unter Beratung durch eine Psychologin. Die Ausprägung von Nebenwirkungen wurde über eine ordinale numerische Rating-Skala mit Werten zwischen 0 (trifft nicht zu) und 10 (trifft voll zu) abgefragt [7]. Weitere wesentliche Datenpunkte waren Alter, Geschlecht und Berufsgruppe. Die Arbeitsfähigkeit in den Folgetagen der Injektionen wurde ebenfalls durch Selbstangabe erhoben. Es folgten weitere Fragen, z. B. Beispiel zu ärztlichen Konsultationen, zur Akzeptanz der Impfung allgemein, und es wurde die Möglichkeit einer Freitext-Rückmeldung angeschlossen. Anschließend wurde der Fragebogen als *Online*-Modul sowohl für das Browser-basierte Intranet und als Modul für die klinikeigene „Meine Uniklinik“-App als auch für die studentische Evaluationsplattform „Evasys“ formatiert. In einem Pretest mit 10 Personen wurde überprüft, ob alle Antwortmöglichkeiten berücksichtigt worden waren und ob die Fragen und Antwortmöglichkeiten einfach und verständlich waren. Anschließend wurde beurteilt, ob in Zahlenwerten wirklich das abgebildet wurde, was die Prätest-Teilnehmer auch aussagen wollten. Die Datensätze des Prätests wurden nicht in die Gesamtanalyse inkludiert. Durch eine Fangfrage wurden unplausible Datensätze identifiziert und von der Analyse ausgeschlossen. Zur Validierung wurde der finale Gesamtdatensatz zufällig geteilt und die zentralen Tendenzen der Items miteinander verglichen. Dabei ergaben sich keine Unterschiede. Studierende der Humanmedizin der Albert-Ludwigs-Universität Freiburg konnten vom 24.03.2021 bis 07.04.2021 an der Umfrage teilnehmen. MitarbeiterInnen des Universitätsklinikums Freiburg konnten den Fragebogen zwischen dem 19.03.2021 und dem 31.03.2021 online beantworten. Dieser konnte mit den persönlichen Login-Daten jeweils nur einmal ausgefüllt werden. Die unberechtigte Teilnahme wurde durch eine Plausibilitätsfragestellung erschwert. Die Datensätze wurden ohne Verbindung mit den Login-Daten in einer Datenbank gespeichert und anschließend als „Excel“-Tabelle (Microsoft, Redmond, Washington, USA) ausgegeben und weiter prozessiert.

Die statistische Analyse erfolgte mit IBM SPSS Statistics 27 (SPSS [Statistical Package for the Social Sciences] für Windows, V.27; SPSS Inc, Chicago, IL USA). Für Vergleiche zwischen einzelnen Gruppen wurden für nichtparametrische ordinale Daten der Kruskal-Wallis-Test und – wenn indiziert – der Mann-Whitney-Test verwendet. Als Signifikanzwert für Unterschiede wurde ein *p*-Wert <0,001 angenommen. Zur Vermeidung einer Alphafehler-Kumulierung bei multiplen Vergleichen wurde eine Bonferroni-Korrektur durchgeführt.

## Ergebnisse

Insgesamt 1267 MitarbeiterInnen und 775 Studierende haben den Fragebogen innerhalb des Observationszeitraums online beantwortet. 54 Datensätze wurden wegen nichtplausibler oder unvollständiger Angaben ausgeschlossen, die finale Stichprobe bestand aus 1988 Befragten. Das durchschnittliche Alter lag bei 37,13 Jahren (Standardabweichung: 13,73). 69,8 % der Befragten waren weiblich, 48,1 % gehörten zu therapeutischen und technischen Berufsgruppen mit Patientenkontakt oder mit anderen Risikokonstellationen, 38 % waren Studierende, 10,6 % waren dem pflegerischen und 4 % dem ärztlichen Dienst zuzuordnen.

**Figure 1:**
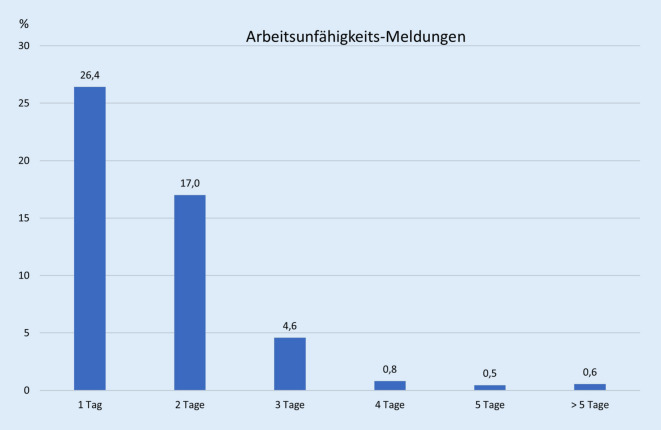

Nach der Impfung fühlten sich nur 18 % der Befragten unmittelbar wieder arbeitsfähig. 51 % aller Befragten mussten sich sogar nach der Impfung für mindestens einen Tag arbeitsunfähig melden (Abb. 1). Geschlechterspezifische oder altersabhängige Unterschiede bestanden bezüglich der Dauer der Arbeitsunfähigkeit nicht.

**Figure 2:**
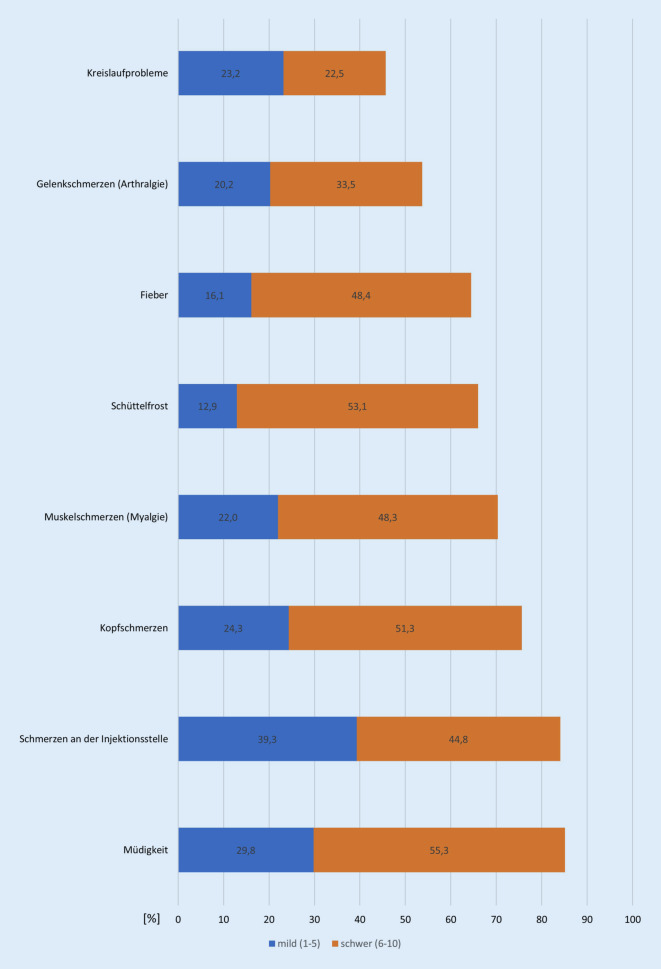

Lokale und systemische Nebenwirkungen waren häufig. Nur 14,4 % der Befragten gaben an, die Impfung ganz ohne relevante Nebenwirkungen vertragen zu haben. Häufigste Nebenwirkung war Müdigkeit, gefolgt von Schmerzen an der Injektionsstelle. In absteigender Häufigkeit folgten Kopfschmerzen, Gliederschmerzen und Schüttelfrost (Abb. 2). Männliche Befragte gaben die Nebenwirkungen jeweils in statistisch signifikant stärkerer Ausprägung an. Die Schwere der Nebenwirkungen nahm mit zunehmendem Alter signifikant ab, dementsprechend war die Dauer bis zur selbstempfundenen Arbeitsfähigkeit bei den jüngeren Befragten etwas länger (Tab. 1).

**Table 1:** 

			Skala 0–10; 0 trifft gar nicht zu, 10 trifft voll zu										Wann wieder arbeitsfähig gefühlt	Tage arbeitsunfähig
			1. Impfdosis und Ausprägung der Nebenwirkungen								Erste Dosis der Impfung wurde gut vertragen	Ich würde mich wieder für Impfung entscheiden		
			Schmerzen Injektionsstelle	Müdigkeit	Kopfschmerzen	Muskelschmerzen (Myalgie)	Schüttelfrost	Gelenkschmerzen (Arthralgie)	Fieber	Kreislaufprobleme				
	*n*	%	Mittelwerte (Skala 0–10)									Mittelwerte		
Gesamt	1988	100	4,90	5,54	5,19	4,79	5,17	3,47	4,85	2,53	5,31	9,05	2,86	1,75
*Geschlecht*														
w	1389	69,87	4,56	4,83	4,60	4,26	4,95	2,82	4,67	1,78	5,23	9,11	2,88	1,57
m	599	30,13	5,04	5,85	5,45	5,02	5,27	3,75	4,93	2,85	5,34	9,03	2,81	1,84
Mann-Whitney-Test	p‑Wert		0,005	<0,001	<0,001	<0,001	0,054	<0,001	0,159	0,001	0,460	0,763	0,919	0,505
*Alter (Jahre)*														
≤25	608	30,58	6,15	6,54	6,48	6,10	6,92	4,27	7,07	3,82	4,92	9,27	2,86	1,98
26–35	467	23,49	5,65	5,95	6,04	5,67	6,04	4,00	5,74^*^	2,78^*^	5,49	9,11	2,6^*^	1,54
36–45	279	14,03	4,80^*^	5,22^*^	4,50^*,**^	4,17^*,**^	4,85^*,**^	3,02^*,**^	3,95^*,**^	1,72^*,**^	5,65	8,89	2,57^*,**^	1,66
46–55	342	17,20	3,56^*,**^	4,78^*,**^	4,17^*,**^	3,63^*,**^	3,40^*,**^	2,86^*,**^	2,80^*,**^	1,57^*,**^	5,47	8,79	2,53	1,61
>55	292	14,69	2,74^*,**^	4,02^*,**^	3,02^*,**^	2,58^*,**^	2,55^*,**^	2,10^*,**^	2,10^*,**^	1,34^*,**^	5,28	9,01	2,62	1,76
Mann-Whitney-Test			^*^P < 0,001 vs. ≤ 25 Jahre; ^**^P < 0,001 vs 26–35 Jahre											
*Berufsgruppe*														
StudentIn	757	38,1	6,31	6,58	6,61	6,26	6,96	4,31	7,00	3,56	5,08	9,32	2,79	1,86
Arzt/Ärztin	65	3,3	4,11	5,28	4,02	4,69	3,75	2,66	3,32	1,03	5,57	8,97	2,34	1,36
Pflegedienst	210	10,56	4,04	4,67	4,08	4,16	4,35	3,65	4,41	2,14	5,25	8,95	2,69	1,53
Sonstige	956	48,1	4,02	4,93	4,40	3,76	4,04	2,83	3,35	1,90	5,47	8,88	2,58	1,71

**Figure 3:**
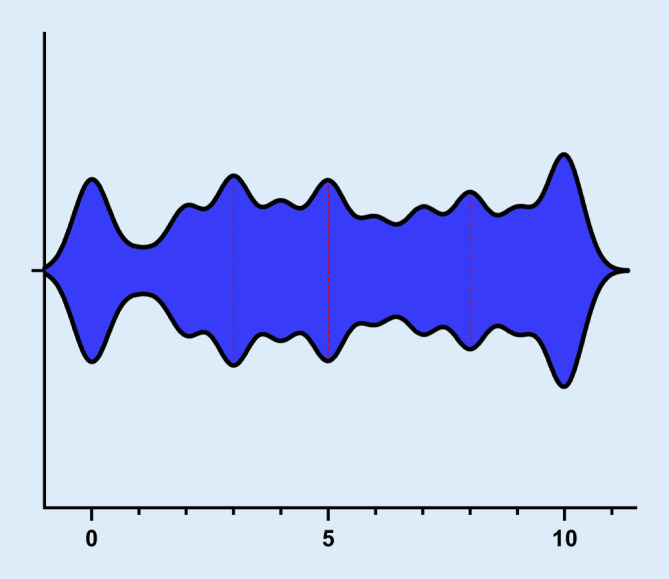

Insgesamt 56 % der Befragten gaben retrospektiv an, die Impfung eher gut (Werte 5–10 auf der Skala) vertragen zu haben (Abb. 3). Bezüglich der selbstempfundenen Verträglichkeit konnten keine Unterschiede zwischen den einzelnen Alters- oder Berufsgruppen ausgemacht werden.

Wegen der Nebenwirkungen ärztlichen Rat eingeholt haben 73 Personen (3,7 %), 61 Geimpfte (3,1 %) gaben an, Symptome bemerkt zu haben, die sie an eine Sinusvenenthrombose haben denken lassen („Krampfanfall, Doppelbilder, Nackensteife, fokale Ausfälle wie Paresen etc.“). Es wurde jedoch keine lebensbedrohliche Nebenwirkung (Sinusvenenthrombose, Anaphylaxie o. ä.) im Zusammenhang mit der Impfung beobachtet oder später an den betriebsärztlichen Dienst gemeldet.

Von den Befragten würden sich 90 % wieder für eine Impfung mit ChAdOx1‑S entscheiden.

## Diskussion

In dieser Studie wurden Nebenwirkungen und Arbeitskraftausfälle in einer deutschen Universitätsklinik durch anonyme Befragung von Geimpften erhoben. Über die Hälfte der Geimpften musste sich für mindestens einen Tag arbeitsunfähig melden. Die durchschnittliche Krankheitsdauer betrug 1,75 Tage, jedoch fühlten sich die Geimpften im Schnitt erst nach fast 3 Tagen wieder völlig arbeitsfähig. Diese Differenz lässt darauf schließen, dass die Impfungen eventuell vor einem freien Tag stattgefunden haben oder dass MitarbeiterInnen wieder zur Arbeit gekommen sind, obwohl sie sich noch nicht wieder vollständig gesund gefühlt haben. Dies muss insbesondere beachtet werden, wenn frisch Geimpfte an Risikoarbeitsplätzen eingesetzt sind. Selbstberichtete Nebenwirkungen nach der Immunisierung mit ChAdOx1‑S waren mit 85 % nach der ersten Dosis häufig.

Dabei spielten vor allem lokale Nebenwirkungen wie Schmerzen an der Injektionsstelle eine große Rolle. Systemische Wirkungen wie Müdigkeit, Kopfschmerzen, gastrointestinale Beschwerden und Schüttelfrost sind am ehesten die Folge der Immunantwort und waren häufiger ausgeprägt als in der Phase-III-Zulassungsstudie [3], was sich eventuell mit dem geringeren Durchschnittsalter der Probanden in unserer Stichprobe erklären lässt. Insgesamt war zu verzeichnen, dass die Ausprägung der Nebenwirkungen von männlichen sowie von jüngeren Befragten stärker angegeben wurde.

Neben den definiert abgefragten typischen Nebenwirkungen wurden im Freitext Lymphknotenschwellungen, Schlafstörungen, Konzentrationsstörungen, Exantheme und gastrointestinale Symptome angegeben. All diese Nebenwirkungen sind auch für andere Impfungen wie die Immunisierung gegen Influenza o. ä. beschrieben und repräsentieren im Allgemeinen die unspezifische Immunantwort.

Bis zum 31.08.2021 wurden in Deutschland 12.645.915 Impfungen mit ChAdOx1‑S durchgeführt. In diesem Zusammenhang wurden 41.534 Verdachtsfälle auf unerwünschte Nebenwirkungen an das Paul-Ehrlich-Institut gemeldet, davon in 4465 Fällen mit schwerwiegenden Reaktionen. Als schwerwiegende Reaktionen gelten solche, bei denen die Personen im Krankenhaus behandelt werden oder Reaktionen, die als medizinisch bedeutsam eingeordnet wurden [8]. Schwere Nebenwirkungen mit Notwendigkeit zur Hospitalisierung infolge der Impfung wurden von keinem der 1988 Geimpften in unserer Erhebung angegeben.

In sehr seltenen Fällen treten nach der Impfung mit ChAdOx1‑S Sinusvenenthrombosen auf. Es handelt sich um Immunothrombosen durch Aktivierung der Thrombozyten über irreguläre Antikörper gegen Plättchenfaktor IV. Durch Nachweis dieser Antikörper und durch Ausschluss einer heparininduzierten Thrombozytopenie (HIT), die dem klinischen Bild ähneln kann, wird die Diagnose des Thrombose-mit-Thrombozytopenie-Syndroms (TTS) gestellt [9]. In seltenen Fällen verläuft diese schwerwiegende Nebenwirkung tödlich. Die Therapie besteht in der Antikoagulation sowie der Gabe von hoch dosierten intravenösen Immunglobulinen (IVIG) in einer Dosierung von 1 g/kg Körpergewicht pro Tag an zwei aufeinanderfolgenden Tagen [10]. In unserer Stichprobe wurde diese Nebenwirkung nicht beobachtet. Jedoch ist sie durch die mediale Begleitung sehr bekannt und hat zu zahlreichen verunsicherten Rückfragen der Befragten an die Studienleiter geführt. Die mediale Begleitung der vergleichsweise seltenen Nebenwirkung hat möglichweise den Vektorimpfstoff unverhältnismäßig diskreditiert und der Akzeptanz der COVID-19-Impfung an sich geschadet. In einer französischen Studie beispielsweise zeigte über ein Viertel aller Beschäftigten im Gesundheitssystem eine Skepsis oder vollständige Ablehnung gegenüber COVID-19-Impfstoffen. Diese wurde durch die mediale Aufbereitung der seltenen spezifischen Nebenwirkungen von ChAdOx1‑S dramatisch verstärkt. Die Ablehnung war größer bei Berufsgruppen mit schlechterem medizinischem Fachwissen [11].

Etwa 4 % der Teilnehmenden gaben an, wegen Nebenwirkungen einen Arzt konsultiert zu haben. Dies mag vor dem Hintergrund eines sehr reaktiven und wirksamen Pharmakons zunächst nicht erstaunen, dass aber über 3 % der Geimpften ihre Symptome selber mit einer Sinusvenenthrombose in Verbindung brachten, reflektiert die Verunsicherung durch die hohe mediale Präsenz dieser Komplikation im Observationszeitraum.

Obwohl alle Geimpften sich freiwillig für die Immunisierung mit ChAdOx1‑S entschieden hatten, gaben immerhin 161 (8,1 %) nur Werte zwischen 0–5 auf einer Skala von 0–10 (0: trifft gar nicht zu) auf die Frage an, ob sie sich wieder für eine Impfung mit diesem Impfstoff entscheiden würden. Mit über 90 % sind jedoch die Befürworter in einer klaren Mehrheit. Hierbei spielte es keine Rolle, ob die Nebenwirkungen stark oder weniger stark erlebt wurden.

## Limitationen

Eine einheitliche Dokumentation über die gesamte Population aller geimpfter MitarbeiterInnen wurde wegen Bedenken der zuständigen Ethikkommission und der Personalvertretung nicht angefordert: Insbesondere sollte verhindert werden, dass der Arbeitgeber über eine „Impfliste“ verfügt, um das Vertrauen in die Möglichkeit zur freien Entscheidung pro oder wider Impfung bei den MitarbeiterInnen und Studierenden zu erhalten. Somit kann nicht mit absoluter Sicherheit berechnet werden, ob die mit 1988 Befragten große Stichprobe völlig repräsentativ für die gesamte Population aller Geimpften in diesem Zeitraum ist. Unter Umständen haben MitarbeiterInnen, die keine Nebenwirkungen verspürt haben, weniger Bereitschaft gezeigt, an der Umfrage teilzunehmen („Reporting Bias“). Durch die Befragung von medizinischem Fachpersonal bei den Teilnehmern könnte eine „Observer Bias“ hinsichtlich einer möglichen Häufung von Nebenwirkungen bestehen.

## Fazit für die Praxis

Mit Impfstoffen wie ChAdOx1‑S stehen potente Mittel zur Eindämmung der SARS-CoV-2-Pandemie zur Verfügung. Bei der Planung von Impfkampagnen bei Berufstätigen sollte beachtet werden, dass sich über 80 % des geimpften Personals in der Selbstangabe nicht am Folgetag wieder arbeitsfähig fühlte und dass über die Hälfte der ArbeitnehmerInnen und Studierenden am Folgetag tatsächlich ausgefallen sind. Stärker von Nebenwirkungen betroffen sind männliche und jüngere Geimpfte. Auf die Dauer der Krankmeldung hatte die Stärke der Nebenwirkungen jedoch keinen Einfluss. Impfkampagnen in Schlüsselbereichen sollten gestaffelt erfolgen, um die Betriebsfähigkeit aufrechterhalten zu können. Bei dem sehr hohen Schutz der Geimpften vor einer SARS-CoV-2-Infektion überwiegen bei hoher Impfquote innerhalb eines Betriebs dennoch die Vorteile durch Verringerung der Transmission und Verhinderung von Krankheitstagen. Bemerkenswert ist, dass sich 90 % der Befragten nach den erlebten Erfahrungen erneut für eine Impfung entscheiden würden.
